# Alcohol and the Intestine

**DOI:** 10.3390/biom5042573

**Published:** 2015-10-15

**Authors:** Sheena Patel, Rama Behara, Garth R. Swanson, Christopher B. Forsyth, Robin M. Voigt, Ali Keshavarzian

**Affiliations:** 1 Department of Internal Medicine, Division of Digestive Diseases and Nutrition, Rush University Medical Center, Chicago, IL 60612, USA; E-Mails: Garth_Swanson@rush.edu (G.R.S.); Christopher_Forsyth@rush.edu (C.B.F.); Robin_Voigt@rush.edu (R.M.V.); Ali_keshavarzian@rush.edu (A.K.); 2 Department of Biochemistry, Rush University Medical Center, Chicago, IL 60612, USA; 3 Department of Pharmacology, Rush University Medical Center, Chicago, IL 60612, USA; 4 Department of Molecular Biophysics & Physiology, Rush University Medical Center, Chicago, IL 60612, USA; 5 Division of Pharmacology, Utrecht Institute for Pharmaceutical Sciences, Faculty of Science, Utrecht University, Utrecht PO Box 80125, The Netherlands

**Keywords:** alcohol, dysbiosis, endotoxemia, gut leakiness

## Abstract

Alcohol abuse is a significant contributor to the global burden of disease and can lead to tissue damage and organ dysfunction in a subset of alcoholics. However, a subset of alcoholics without any of these predisposing factors can develop alcohol-mediated organ injury. The gastrointestinal tract (GI) could be an important source of inflammation in alcohol-mediated organ damage. The purpose of review was to evaluate mechanisms of alcohol-induced endotoxemia (including dysbiosis and gut leakiness), and highlight the predisposing factors for alcohol-induced dysbiosis and gut leakiness to endotoxins. Barriers, including immunologic, physical, and biochemical can regulate the passage of toxins into the portal and systemic circulation. In addition, a host of environmental interactions including those influenced by circadian rhythms can impact alcohol-induced organ pathology. There appears to be a role for therapeutic measures to mitigate alcohol-induced organ damage by normalizing intestinal dysbiosis and/or improving intestinal barrier integrity. Ultimately, the inflammatory process that drives progression into organ damage from alcohol appears to be multifactorial. Understanding the role of the intestine in the pathogenesis of alcoholic liver disease can pose further avenues for pathogenic and treatment approaches.

## 1. Introduction

Alcohol abuse is a significant contributor to the global burden of disease and can lead to tissue damage and organ dysfunction in a subset of alcoholics. For example, approximately 20%–30% of heavy drinkers develop clinically significant alcoholic liver disease (ALD) including alcoholic steatohepatitis and cirrhosis [1]. This observation indicates that although alcohol consumption is necessary it is not sufficient to cause clinically relevant organ damage [2,3]. Several factors have been shown to influence the progression of alcohol-related diseases, including duration of alcohol abuse, gender, obesity [4], ethnicity, comorbidities and pre-existing underlying liver disease such as hepatitis C infection (HCV) and hemochromatosis [5,6,7]. Non-alcoholic fatty liver disease (NAFLD) associated with obesity/metabolic syndrome also has several pathological features in common with ALD including microbiota dysbiosis and alcohol production by intestinal bacteria and can synergize with ALD if chronic alcohol consumption is added [8,9,10,11,12,13,14,15,16]. Chronic alcohol use also has disruptive effects on growth factor, cytokine and immune function that can result in greater risk of infections as well as immune dysregulation contributing to inflammation in ALD [17,18,19]. However, a subset of alcoholics without any of these predisposing factors can develop alcohol-mediated organ injury [20]. Alcohol-induced organ damage occurs via an inflammatory process, thus it is crucial to identify the source of inflammation that is contributing to disease progression.

The gastrointestinal tract (GI) could be an important source of inflammation in alcohol-mediated organ damage. The GI harbors the largest and most complex microbial community in the body including pro-inflammatory triggers such as endotoxins (*i.e.*, lipopolysaccharide, LPS) [21,22]. Alcohol consumption can elicit systemic pro-inflammatory changes via two GI tract-mediated mechanisms: (1) changing the intestinal microbiota composition and/or function (*i.e.*, dysbiosis) resulting in increased production of LPS and/or (2) disrupting intestinal barrier integrity (*i.e.*, intestinal hyperpermeability) permitting the passage of luminal lipopolysaccharide (LPS) into the systemic circulation. Studies indicate that alcohol-induced bacterial translocation is due to pro-inflammatory cytokine release as a consequence of immune activation by LPS, a critical component of the outer membrane of Gram-negative bacteria [23,24]. In fact, there is a direct association between ethanol administration and increased plasma LPS levels in animals [25]. TLR4 (Toll-like receptor 4) has a vital role in the immune response to bacterial translocation by binding to LPS and initiating a cascade of activation of Kupffer cells and macrophages [26,27]. TL4 has been proven to have a role in liver disease, as it is one of several genes linked to elevated risk of developing cirrhosis in patients with chronic Hepatitis C [28]. Indeed, pro-inflammatory cytokines (e.g., tumor necrosis factor alpha, TNFα) are elevated in the terminal ileum of alcohol-fed mice [29]. Furthermore, an important role for endotoxin in the development of alcohol-induced tissue injury is evident based on studies showing that administration of substances that alter the intestinal bacteria including antibiotics or probiotics attenuate alcohol-induced damage such as liver disease [30,31,32,33].

Seventy percent of the liver’s blood supply comes from the portal vein making the liver continuously exposed to intestine-derived nutrients as well as intestinal bacteria and bacterial components. Indeed, LPS levels are increased in the portal and systemic circulation following alcohol intake [34,35]. Both Kupffer cells and macrophages within the liver respond to LPS via a myriad of mechanisms including activation of the TLR4 eliciting the production of reactive oxygen species (ROS), leukotrienes, chemokines, as well as cytokines. The production of these factors results in tissue inflammation and contributes to alcohol-induced organ pathology.

In this review, we will provide an overview of mechanisms of alcohol-induced endotoxemia (*i.e.*, dysbiosis and gut leakiness) and highlight the predisposing factors (e.g., disrupted circadian rhythms) for alcohol-induced dysbiosis and gut leakiness to endotoxins.

## 2. Alcohol-Induced Intestinal Dysbiosis

The gut microbiota refers to the collection of bacteria in the gastrointestinal tract (GI) [36,37]. Bacterial communities differ from one individual to the next due to age, dietary habits, medications, illness, stress, and geographical origin just to name a few [38,39]. The host and the intestinal microbiota share a symbiotic relationship and the host benefits greatly from the microbiota including (but not limited to) catabolism of dietary fiber, vitamin synthesis, maintenance of the intestinal barrier, preventing the growth of pathogenic bacteria, and the microbiota can even influence behavior of the host [36,39,40,41,42,43].

Alcohol consumption alters the intestinal microbiome [3,30,44,45,46,47,48]. Although the changes are specific to the species being examined (e.g., mouse, rat, human) and the alcohol consumption protocol used (e.g., chronic *versus* acute) there is a trend for an increase in pro-inflammatory bacteria following exposure to alcohol. Alcoholics have lower abundance of bacteria from the phylum Bacteriodetes and butyrate producing bacteria (generally believed to be anti-inflammatory) and greater bacteria from the phylum Proteobacteria (generally believed to be pro-inflammatory [3,48]. Short chain fatty acids (SCFA) are produced by certain bacteria and are known to influence intestinal barrier integrity [49,50,51,52].

Similar findings are observed in the fecal microbiota of cirrhotic subjects, with a reduction in Bacteroidetes and an increase in Proteobacteria compared to healthy controls [53]. This is important as an increase in pro-inflammatory bacteria can have substantial deleterious effects in the periphery including an inflammatory immune response. Pro-inflammatory cytokines are released as a consequence of LPS binding to TLR4 [23,24] particularly in Kupffer cells and macrophages [26,27]. Indeed, pro-inflammatory cytokines (e.g., tumor necrosis factor, TNF) are elevated in the terminal ileum of alcohol-fed mice [29]. Particularly TNF is elevated in the lamina propria in an *in vivo* model of alcoholic liver with intestinal dysbiosis [54]. It should also be noted that the intestinal microbiota also influences the immune phenotype that could in turn impact organ damage [55,56,57].

In addition to changes in the intestinal bacteria population, bacterial overgrowth can also occur as a consequence of alcohol consumption. Mice fed alcohol for three weeks have changes consistent with intestinal bacterial overgrowth [45]. The reason is unclear but a theory suggests that alcohol may decrease the intestinal motility resulting in an increase in luminal bacteria. Other alternatives may be from impaired bile production, dysmotility, or altered gastric pH [58]. Regenerating islet-derived protein 3 gamma (Reg3g) is a bactericidal protein secreted from Paneth cells and intestinal epithelial cells that can regulate bacterial overgrowth. Reg3g is suppressed in the small intestine after alcohol consumption [45].

In summary, intestinal dysbiosis caused by alcohol can exacerbate the detrimental effects associated with alcohol. Changes such as altered immune phenotype due to dysbiosis, increased proinflammatory bacteria (e.g., gram negative bacteria containing LPS), a reduction in SCFA-producing bacteria, or bacterial overgrowth would be expected to negatively impact the host via multiple mechanisms including intestinal barrier integrity.

## 3. Barrier Function and Alcohol-Induced Intestinal Hyperpermeability

The intestine is the largest interface between the host and the environment and the integrity of the intestinal barrier is necessary to separate pro-inflammatory luminal contents from the systemic circulation [59]. Key components of the intestinal barrier include an immunological barrier and a biochemical/physical barrier and together these components regulate the passage of luminal factors such as food antigens, bacteria, and bacterial products into the intestinal mucosa and subsequently into the portal and systemic circulation. An intact and healthy barrier is essential to maintain a healthy state. Factors that disrupt these components of normal barrier function could promote local and systemic inflammation that could lead to tissue injury and organ damage [59,60].

### 3.1. Immunologic Barrier

Secretary IgA is amongst the most abundant class of antibodies found in the intestinal lumen and it protects the intestinal epithelium from enteric toxin and pathogenic damage [61,62,63]. Indeed, IgA appears to exert its anti-inflammatory effects by reducing bacterial pro-inflammatory pathways and limiting LPS-induced cytokine release (e.g., IL1 and TNFα). Several studies have shown that IgA level is increased in alcoholics which might be a compensatory protective mechanism for limiting alcohol-induced damage [61,64,65]. Since intestinal IgA levels are not decreased, it does not appear that alcohol-induced disrupted intestinal barrier function is a consequence of abnormal intestinal IgA and immunologic barrier function.

### 3.2. Biochemical/Physical Barrier

Key components of the non-immunologic intestinal barrier include an unstirred water layer, mucosal surface hydrophobicity, surface mucous coat, endothelial factors [59], and epithelial factors (most importantly tight junctions). Thus, alcohol can cause gut leakiness by impacting any of these components of intestinal barrier function. For example, alcohol effects on mucus could potentially induce gut leakiness. Indeed, alcohol affects MUC-2 protein expression [66], which is one of the key components of intestinal mucus layer [67]. Other potential means for alcohol to cause gut leakiness is to increase trans-epithelial passage of molecules. Since it is well-established that alcohol can increase cellular membrane fluidity [68], it is plausible that alcohol abuse results in disrupted intestinal epithelial cell membrane fluidity leading to gut leakiness. While these factors are all important, components of the biochemical/physical barrier, the epithelial layer of intestinal barrier may very well be the most important factor in mediating barrier integrity. The epithelial layer is a highly selective barrier that allows the absorption of nutrients from the intestine lumen into the circulation and restricts the passage of toxic compounds (e.g., endotoxin). The two major routes of epithelial permeation are transepithelial and paracellular pathways and while the physiology of these tightly regulated pathways is incompletely understood it is clear that epithelial permeation is differentially regulated by various physiologic and pathologic conditions [69,70,71,72]. For example, barrier function is often compromised in patients with Crohn’s disease [73] and other intestinal diseases mediated by inflammation and/or infection [74,75,76]. The intestinal barrier has also been shown to be impacted in alcoholics with liver disease [2,77,78].

Tight junctions seal the space between adjacent epithelial cells and therefore regulate barrier function [22,72]. The tight junctions are made up of a complex network of proteins including occludin, claudins, and the junctional adhesion molecule (JAM), which interact with the actin cytoskeleton via proteins such as ZO-1 [72,79,80]. Each of these components is critical for proper functioning of the barrier and many of these components are often impacted by alcohol. Exposure of intestinal epithelium to actin-depolymerizing drugs disrupts barrier function [81] and disruption of the actin cytoskeleton exaggerates alcohol-induced disruption of the intestinal barrier *in vitro* (*i.e.*, intestinal epithelial cell monolayers) [82,83]. Duodenal biopsy samples from cirrhotic patients demonstrate enlarged intercellular spaces below the tight junctions, indicating morphological changes that persist with long-term alcohol use [84]. In addition, alcohol consumption is associated with a decrease in ZO-1 in rodents and in alcoholic patients [85,86,87].

Caco-2 intestinal epithelial cells have been widely used to study intestinal permeability *in vitro* and have been very useful in elucidating the molecular mechanisms underlying alcohol-induced intestinal barrier dysfunction. Exposure of Caco-2 cells to alcohol causes a myriad of effects resulting in impaired barrier function including: (1) induction of nitric oxide and increased oxidative stress burden (in part mediated though NF-kB and PKC) [82,83,88]; (2) calcium release and MAP kinase activation [89,90]; (3) depletion of zinc [91,92]; (4) activation of myosin light chain kinase (MLCK) [93]; and (5) microRNA 212 (miR-212) down-regulation of ZO-1 [86,87]. Metabolism of alcohol to acetaldehyde has also been shown to promote leakiness of Caco-2 monolayers via a phosphatase-related mechanism [94] as well as a Snail-related mechanism [95]. Overall, many *in vitro* studies support the finding that alcohol promotes increased intestinal permeability *in vitro* through multiple pathways [96].

The revelation that alcohol promotes increased intestinal permeability *in vitro* is supported by *in vivo* research. There are several well-established methods to assess intestinal permeability *in vivo*. All methods are based on oral administration of poorly/non-metabolized and poorly absorbed markers that are secreted by the kidney allowing for easy measurement of their urinary content as indication of intestinal barrier integrity. Although other methods exist, the predominant method employs the use of polysaccharides like sucrose, mannitol, lactulose, and sucralose [75,76,97]. The advantage of using these sugars is that they allow for approximating the site of the barrier dysfunction in the GI tract. For example, urinary sucrose primarily represents gastroduodenal permeability as oral sucrose is rapidly absorbed and broken down by brush border enzymes, urinary mannitol represents barrier dysfunction in the proximal small bowel, and urinary lactulose indicates leakiness of the small bowel as lactulose is fermented by colonic bacteria [75,97]. In contrast, intestinal bacteria are not capable of fermenting sucralose and urinary sucralose represents barrier integrity throughout the entire intestine. Using this method, alcohol consumption has been well-established to cause intestinal hyperpermeability in mice and rats [25,98] and this is associated with a reduction in the levels of the messenger RNA [19] and proteins [85,98] of tight junction components necessary for maintaining barrier integrity.

Micro-RNAs are small noncoding RNAs that regulate numerous biological functions including inflammation. Both miR-212 and miR-155 can down-regulate components of the tight junction including ZO-1 [87,99]. The production of reactive oxygen species (ROS) and oxidative damage is a feature associated with monolayer barrier dysfunction *in vitro* and ROS also appear to be critically important *in vivo*. Cytochrome P450 isoform E1 (Cyp2e1) is an important mediator in alcohol metabolism and alcohol metabolism by Cyp2e1 produces ROS [100]. Cyp2e1 is expressed in the small intestine and colon of rodents and humans and is up-regulated in intestinal tissue by chronic alcohol administration [101,102]. One mechanism by which Cyp2e1 increases intestinal permeability is through a circadian clock mechanism [101], which will be further discussed below.

These studies in rodents translate well to human studies. Research has demonstrated that at least a subset of alcoholics develop intestinal hyperpermeability [2,103], which appears to align with the observation that only a subset of alcoholics develop alcoholic-induced liver pathology. Indeed, intestinal hyperpermeability has been reported in human alcoholic subjects [21]. Interestingly, a subsequent study found that intestinal permeability was elevated only in alcoholic subjects with liver disease and not in alcoholics without liver disease [2] suggesting that a “leaky gut” may be crucial for the development of chronic liver disease [2]. Data also demonstrates that increased permeability only occurs in susceptible alcoholics, as a specific mechanism whereby gut-derived substances such as endotoxin and N-formylmethionyl-leucyl-phenylalanine (fMLP) can enter into the portal venous circulations [2,104,105]. Alcohol-induced gut leakiness in humans also appears to be due to disruption of tight junctional proteins like ZO1 [86,87].

## 4. Environmental Co-Factors for Alcohol-Induced Dysbiosis and Barrier Dysfunction

A number of factors can influence the impact of alcohol on the intestine and one that is of particular interest is how circadian rhythms interact with and/or are contributing to alcohol-induced organ pathology. Circadian rhythms are 24 h biological patterns that synchronize an organism with the daily environmental patterns (e.g., light:dark, eating patterns) and are based on the function of the molecular circadian clock with is a negative feedback loop that takes approximately 24 h to complete [106]. Circadian clock genes are expressed throughout the body, including in the gastrointestinal tract [107]. These genes help regulate colonic motility, nutrient absorption and cell proliferation [108]. Of note, disruption of circadian rhythms has been shown to be a mechanism underlying many inflammatory mediated conditions, such as malignancy, obesity, cardiovascular disease and intestinal disorders [109,110]. Interestingly, alcohol increases the expression of circadian clock proteins (Clock, Per2) in Caco-2 cells and mice and blocking Clock and Per2 by siRNA prevents alcohol-induced hyperpermeability *in vitro* [111]. Subsequent studies show that the up-regulation in Clock/Per2 is due to a ROS-mediated consequence of Cyp2E1 alcohol metabolism [101] indicating that disruption of the molecular circadian clock may be one mechanism influencing alcohol-induced intestinal barrier dysfunction. Interestingly, it has been shown that Cyp2e1 knockout mice exhibit blunted intestinal leakiness and liver inflammation after binge exposure to alcohol [103,112]. Environmental (*i.e.*, disruption of the light:dark cycle) or genetic alterations in the molecular circadian clock (*i.e.*, Clock^∆19^ mutant mouse) increase intestinal permeability in mice [98], exacerbate alcohol-induced intestinal barrier dysfunction [98], and cause intestinal dysbiosis (particularly when combined with a secondary stress like a high-fat, high-sugar diet) [113,114]. Subsequently alcoholic subjects were found to have less total sleep time and increased fragmentation of sleep in addition to significantly lower plasma melatonin levels compared to healthy controls [115], which are indicative of disrupted circadian homeostasis. The lower plasma melatonin levels correlated with increased intestinal permeability and a serum marker of endotoxemia [115].

## 5. Can Alcohol-Induced Changes Be Prevented or Mitigated?

The deleterious effects of alcohol are numerous; however, therapeutic measures can be employed to mitigate alcohol-induced organ damage by normalizing intestinal dysbiosis and/or improving intestinal barrier integrity. Prebiotics are substances that promote the growth/activity of microorganisms [116] and probiotics are microorganisms that are believed to provide health benefits; both function to change the intestinal microbiota profile. Alcohol-induced dysbiosis in rodents can be corrected with beneficial effects on treatment of alcoholic liver disease and by dietary supplementation with either prebiotic oats or probiotic *Lactobacillus GG* [30,31,46,117,118]. Prebiotics have also been shown to restore Reg3G levels to reverse bacterial overgrowth and limit the progression of alcohol-induced steatohepatitis with chronic alcohol feeding [45]. Furthermore, oats and *Lactobacillus GG* supplementation prevents oxidative stress induced by alcohol on the intestinal actin cytoskeleton and tight junctions thereby maintaining intestinal barrier integrity [31,117]. Indeed, probiotics are capable of limiting endotoxemia by modifying intestinal microbiota and improving intestinal barrier function and liver disease in alcohol-fed rodents [31,32,117,118]. These findings were supported by studies from McClain *et al.* in alcohol-fed mice with Lactobacillus GG [30] as well as probiotic therapy of alcoholics [119]. Schnabl *et al.* also reported beneficial effects of long chain fatty acid supplementation in alcohol fed mice [120]. Long chain fatty acids supplementation prevented the decrease in *Lactobacillus* abundance in the stool of alcohol-fed mice and prevented loss of intestinal barrier integrity in these mice [120]. Other studies have also shown that alcohol decreases zinc finger associated protein function in the intestine of alcohol-fed rodents and that zinc supplementation successfully protected the intestine and prevented gut leakiness in alcohol fed rodents [91,92]. Thus, a role exists for therapeutic measures to manage alcohol induced pathologies by mitigating or preventing intestinal dysbiosis and/or barrier dysfunction.

## 6. Conclusions

While alcohol is a necessary component in the development of alcoholic liver disease and cirrhosis, only a subset of alcoholics develop cirrhosis and liver failure [20]. Progression of alcoholic liver disease is driven by an inflammatory process, which appears to be multifactorial. This pro-inflammatory cascade has been supported in multiple studies with emerging evidence suggesting the role of the gut microbiome. Both animal and human studies have suggested that intestine-derived microbial factors and bacterial endotoxins are paramount in promoting the inflammatory process noted in the liver. There is an increasing need to identify mechanisms by which the intestine is contributing to myriad alcohol-induced pathologies to provide new mechanisms for the deleterious effects of alcohol-induced organ damage, but also new avenues for therapeutic options.
